# Soft Tribology and Its Relationship With the Sensory Perception in Dairy Products: A Review

**DOI:** 10.3389/fnut.2022.874763

**Published:** 2022-05-19

**Authors:** Beatriz Corvera-Paredes, Aidee I. Sánchez-Reséndiz, Dora I. Medina, Rosa S. Espiricueta-Candelaria, Sergio Serna-Saldívar, Cristina Chuck-Hernández

**Affiliations:** ^1^Tecnologico de Monterrey, School of Engineering and Sciences, Monterrey, Mexico; ^2^Tecnologico de Monterrey, School of Engineering and Sciences, Atizapán de Zaragoza, Mexico; ^3^Tecnologico de Monterrey, The Institute for Obesity Research, Monterrey, Mexico

**Keywords:** soft tribology, sensory analysis, dairy products, friction coefficient, yogurt

## Abstract

Nowadays, dairy products, especially fermented products such as yogurt, fromage frais, sour cream and custard, are among the most studied foods through tribological analysis due to their semi-solid appearance and close relationship with attributes like smoothness, creaminess and astringency. In tribology, dairy products are used to provide information about the friction coefficient (CoF) generated between tongue, palate, and teeth through the construction of a Stribeck curve. This provides important information about the relationship between friction, food composition, and sensory attributes and can be influenced by many factors, such as the type of surface, tribometer, and whether saliva interaction is contemplated. This work will review the most recent and relevant information on tribological studies, challenges, opportunity areas, saliva interactions with dairy proteins, and their relation to dairy product sensory.

## Introduction

Oral processing, also known as mastication or chewing, is a complex mechanism involving many physical, chemical and biochemical changes with plenty of superficial interactions taking place (1, 2), such as teeth grinding, tongue-palate, tongue-teeth, teeth-food, and tongue-food (3). This is a complex process characterized by a shift from rheology-dominant to tribology-dominant activities being the first process of food consumption that yield energy and essential nutrients to our body (4) and includes all muscle activities, jaw and tonge movements contributing to prepare food for swallowing (1). Oral processing can be defined as the procedure of changing from solid food to a bolus ready to be swallowed, through reducing particle size of the food and mixing them with saliva where mechanical, enzymatic, and even microbiological takes place (4). This process is associated with the description of food quality that all of us made based on the food appearance, flavor, nutrition and texture, being the latter a factor where most of people have an anchored idea based on their past experiences. Texture as already said is related to rheological properties and tribological characteristicas of the food bolus in the different oral processing phases (4), being texture a multidimensional experience perceived during all stages of oral processing. Returning to the beginning to this paragraph, it is important to write that rheology is the study of the deformation of materials, whereas tribology (as described in the following sections) is the science of interacting surfaces in motion and studies lubrication and friction, applied first to study roughness of engineering materials (1).

Dairy products (yogurt, cream, cream cheese) are classified as semi-solid foods because most of them can change their rheological properties depending on the temperature, and one important characteristic is that they normally reside short time during oral processing, exhibiting fluid-dominant attributes, such as thickness: a sensory property highly related to rheological studies. Besides thickness, other attributes perceived commonly in dairy food are creaminess, fattiness, smoothness, stickiness, and astringency, for which classical rheology is not enough to describe them (5). For that reason, many researchers have put their attention on tribology. Recent results have demonstrated the potential of this discipline to predict traits in developing new food products due to the relationship between friction, food composition, and sensory attributes (6).

Owing to the high impact on consumer preferences, the food industry always analyzes sensory perception. The most used equipment to analyze rheological behaviors is the texturometer, the viscometer, and the rheometer. However, these tests are based on shear deformation or destruction of the food, giving a good result on the mechanical properties of the product, but without describing the geometric or surface properties of the sample, which are obtained by rubbing or squeezing the food against some surface. Due to these techniques being insufficient to describe very important characteristics such as creaminess or astringency, the use of tribology and its correlation with sensory analysis has begun to be evaluated (7, 8). Nowadays, there are already some studies of tribological analysis on numerous dairy foods such as yogurt, cream, cream cheese, custards, and others (9–17). The purpose of this review article is to collect and discuss the most relevant and novel information about the tribological analysis of dairy products and the relationship shared by the coefficient of friction (CoF) and the sensory attributes of smoothness, creaminess, and astringency. Finally, we discussed the latest topic in lubrication analysis: adsorption studies.

## Tribology

Tribology can be used to study the complex process of food oral processing, a mechanical function of the human body that undergoes chewing, transportation, and swallowing. The procedures can be divided into three main stages: the first oral phase that relies on the rheological characteristics of food such as brittleness, adhesiveness, and hardness. The second step involves surface and lubrication mechanisms like smoothness and creaminess. The third step is a transitional phase that relies upon tribology factors like thickness, creaminess, and consistency, which may be a critical part of sensory intensity and profile (18).

**Figure 1:**
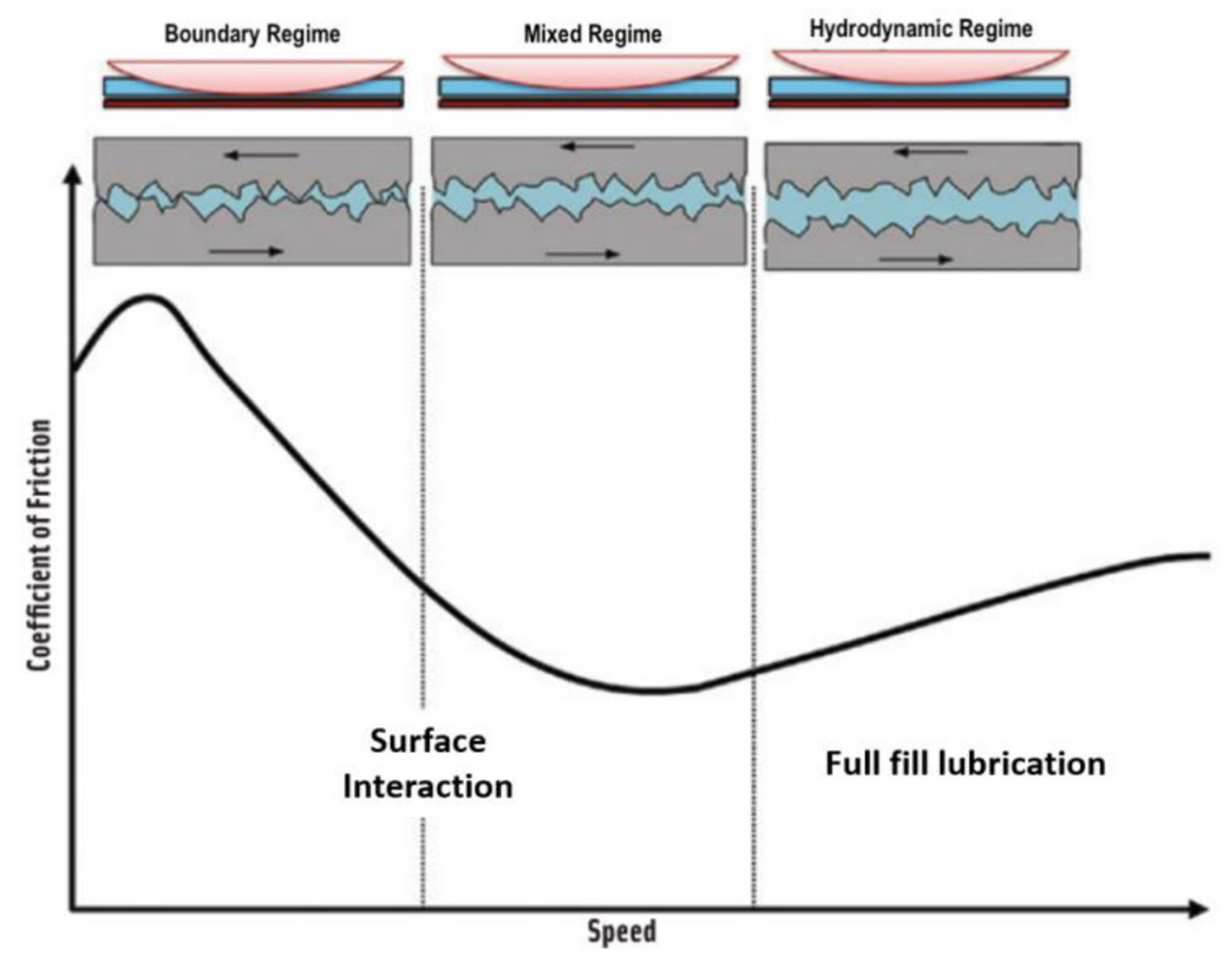
Typical stribeck curve with the three different lubrication regimes. From: Mermelstein (22).

Tribology is defined as the study of lubrication, friction, and wear between two surfaces in relative motion and is commonly used to study mechanical engineering phenomena and materials, oils, and lubricants (6, 19). Several factors influence this parameter, like the surfaces' material, average load, sliding speed, contact area, and temperature. Typically, tribological results are presented as a Stribeck curve (Figure 1), which plots CoF against the sliding speed, and is divided into the following lubrication or friction regimes: (1) *the boundary regime*, where the lower and upper surfaces are in almost complete contact, only separated by a thin layer of lubricant, or in this case food, (2) *the mixed regime*, in which some parts of each surface are still in contact, and (3) *hydrodynamic or fluid regime*, in which the food sample or lubricant withstands the average force applied by surfaces, keeping them completely separated (19, 20). These three regimes represent different food samples between the tongue and palate (21).

Regarding food science, tribology provides information about the CoF resulting from the interactions between food and the surfaces on the oral cavity: tongue, palate, cheeks, and teeth (22). In order to mimic the surfaces within the oral cavity, at least one of the materials in contact with the food is not rigid or, in other words, is soft. These measurements provide insights into several sensory perceptions related to texture, even though obtaining quantitative empirical relationships between frictional interactions of foods with surfaces is challenging. In order to overcome these challenges, the selection of an appropriate tribological system, including instrument, surfaces, food system model, and analysis of the role of saliva, among other variables, is the most relevant element for the use of tribology as an indicator for texture perception (23). Soft tribology is intended primarily to evaluate the geometric properties of a food or beverage during the stages of its consumption. In other words, it aims to explain the relationship between friction properties and food processing in the mouth to describe parameters such as creaminess and smoothness, giving information about consumer preference (19, 23). Nowadays, mouthfeel attributes, such as creaminess, roughness, or astringency commonly found in dairy products, have been linked to friction on the tongue and palate; thus, friction screening tests are increasingly used in product development (22). Despite all the work already reported about oral processing and soft tribology, linking friction characteristics with sensory perception remains a challenge because CoF cannot be defined as a property of the material or food but rather a complex system-dependant related to many factors.

**Figure 2:**
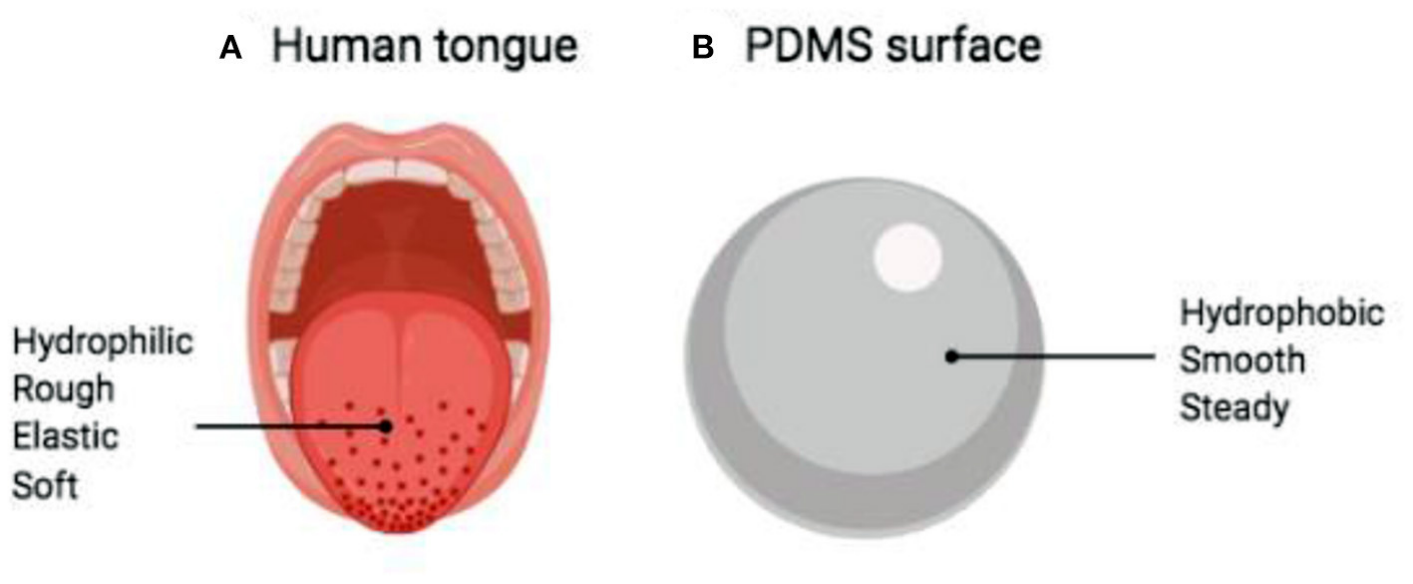
Comparison between **(A)** human tongue and **(B)** untreated PDMS surface. PDMS: Polydimethylsiloxane. Figure edited from Rudge et al. (24).

**Figure 3:**
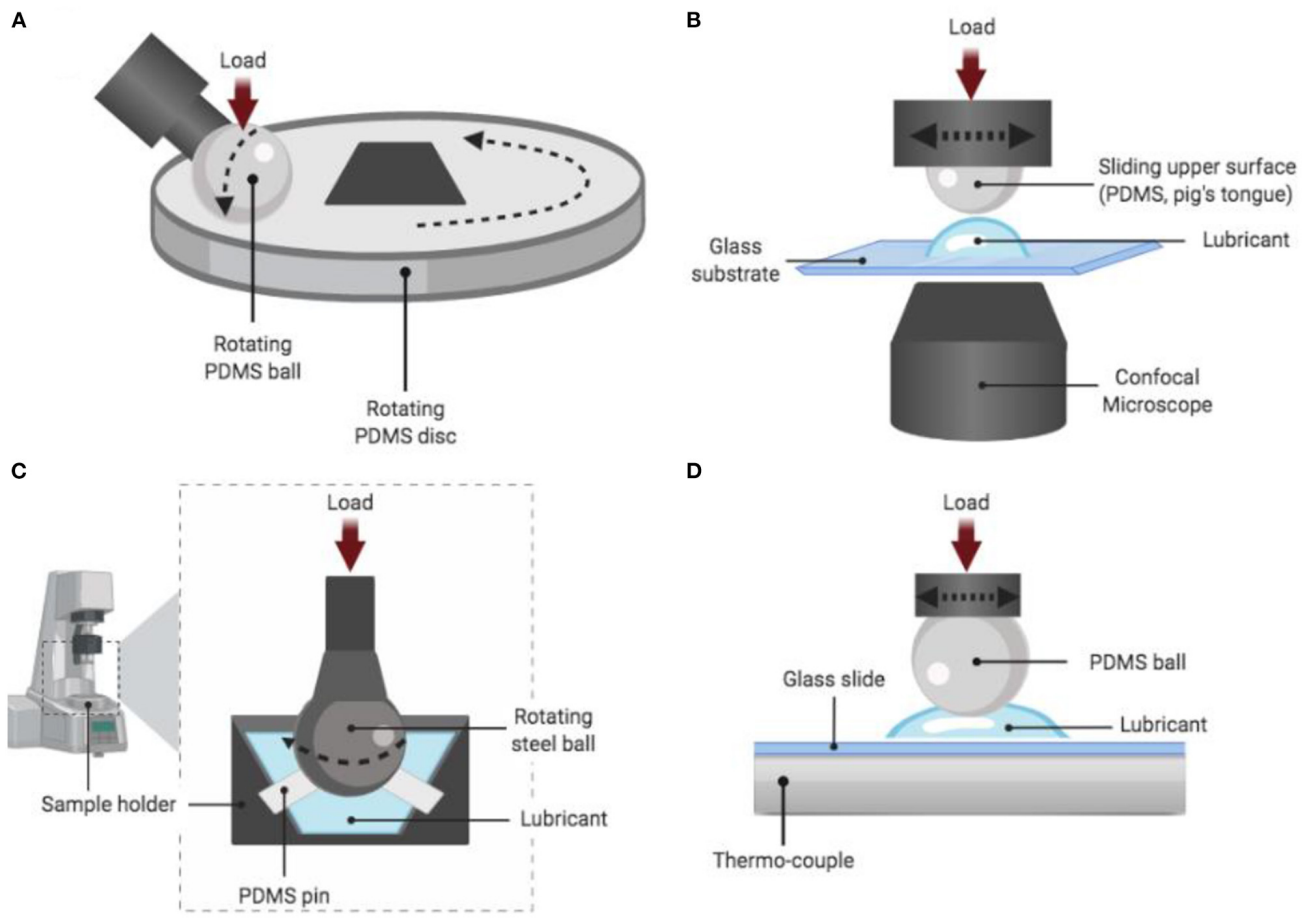
Scheme of the most used equipment for tribological measurement. **(A)** Mini-traction machine in a ball on disk configuration (MTM); **(B)** Optical tribometer configuration (OTC); **(C)** Anton-Paar ball on three pins rheometer attachment and **(D)** High-Frequency Reciprocating Rig (HFRR) device. NOTE: Polydimethylsiloxane (PDMS) is an example of the surface, but other materials can be used. Figure edited from Shewan et al. (6) and Tsui et al. (17).

Among them are the surfaces type used for the test (soft, hydrophilic and elastic like the human tongue, or hard, synthetic and hydrophobic like polydimethylsiloxane -PDMS-, Figure 2), the food properties itself (solid, semi-solid, fluid), the measurement tribometer system or type (being the most commonly used the Mini-Traction Machine (MTM), the Optical Tribometer Configuration (OTC), the Anton-Paar rheometer equipped with a special attachment and the High-Frequency Reciprocating Rig (HFRR), Figure 3, and whether or not saliva interaction is considered in the tribological analysis (23). As mentioned before, the selection of all these factors are important for reliable results in lubrication analysis; however, in this review, we will not discuss them since multiple past works are related to this topic (1, 6, 7, 23–25).

## Principle, Mechanism and Saliva Interactions With Dairy Proteins

Saliva plays an important role in oral processing because helps to wet food, reduce friction in mastication process and influence in the sensory perception of foods (26). The saliva proteins are responsable of the oral lubrication for the food processing and digestion. The main proteins found in saliva are mucins, statherin, prolinerich glycoproteins, acidic protein-rich proteins, and lactoferrin. During oral processing saliva proteins mixes with food to form a bolus which increase the ease of swallowing and give the humans a sensory perception and textural attributes (27). Despite a number of studies addressing the interactions of saliva proteins with polysaccharides, studies related to the interaction with dairy products are still limited. Hence, the interactions between saliva proteins and dairy products are reviewed.

Dairy products contain caseinates and whey proteins like β-lactoglobulin (B-LG), bovine serum albumin, α-lactalbumin, lactoferrin, and immunoglobulins (18). According to Çelebioglu et al. (18), the electrostatic interaction between positively charged whey proteins and negatively charged saliva proteins at low pH, cause astringency for its precipitation around the oral cavity or binding to oral epithelial. Moreover, (28) showed that caseins attract the salivary proteins only *via* non-covalent interactions giving as result the perception of mouth drying. Based on this, the complexity of the dynamic saliva model and dairy proteins, in addition to the variety of parameters such as concentration, pH, heat, and surface properties that effect the friction, the tribology could be a valuable instrument to study the sensory perception.

## Dairy Products, Sensory Perception, and Tribology

Dairy products are among the most studied or preferred foods for tribological analysis. Cream, yogurt and custard are semi-solid foods, defined as oil-in-water emulsions that often contain a dispersed solid phase as proteins (17). Usually, they also contain carbohydrates (lactose and polysaccharides), fat (low or high content) and other soluble components as minerals. These dairy foods require minimum effort for mastication because they promptly form a bolus ready to be swallowed (9). As a result of the rapid swallowing, these foods spend a few seconds in the mouth (29), and therefore they have limited oral interactions consisting just of tongue rolling and swallowing with little or no chewing. As the food contact period with the oral cavity surfaces is short, it is important to pay attention to the flow and the sensations that remain after swallowing, that is, the posterior sensations such as after-mouth feel or astringency. These characteristics will dominate in the tongue of the consumer, who will evaluate the texture of the semi-solid food mainly due to its fluidity and lubricating properties during retention in the mouth (29). In recent years, the relationship between lubrication properties and dairy sensory perception has gained a broad interest in food science and industry over other food types (30, 31). Table 1 summarizes a compilation of the work carried out in recent years on the tribology of dairy products, summarizing the conditions and parameters evaluated, along with the additional studies carried out for obtaining complementary information.

**Table 1:** Summary of tribological studies on dairy products.

**Dairy product**	**Sample composition parameters**	**Tribometer and test**	**Tribometer conditions**	**Surface Material**	**Saliva interaction**	**Additional studies**	**References**
Milk	• Skim milk (0.08% fat) • Milk (1% fat) • Whole milk • (3.5 % fat)	• Discovery hybrid rheometer-3 (TA Instrument, Newcastle, USA) • Double ball on plate	• Load: 1 N • Temperature: 25°C • Sample: 1 ml • Speed: 0.15–750 mm s^−1^	• Upper: 2-polypropylene balls (15.88 mm diameter) • Lower: PDMS disk (40 mm diameter)	Yes	• Rheological • (Viscosity) • Sensory analysis (Astringency) • Microbial (Aerobic plate count and coliform) • Proximate analysis (Lactose, Protein, Fat, Solids)	(5)
Custard	• Starch (1, 2, 3% wt/wt) • κ-carrageenan (0, 0.15, 0.3% wt/wt) • Fat content (0, 3, 6% wt/wt)	Discovery Hybrid Rheometer with ring-on-plate tribo-rheometry (TA Instrument, USA)	• Load: 2 N • Temperature: 35°C • Speed: 0.01 to 6.5 rad s^−1^ for 1 min with 20 points per decade during 10 min.	• Upper: Stainless steel ring • Lower: 3M™ transpore surgical tape	No	• Static light scattering (Particle size) • Rheological (Viscosity and dynamic oscillation) • Sensory analysis (Thickness, Smoothness, Powderiness, Creaminess and Oiliness)	(9)
Milk	• Whole milk (3.6 wt% fat) • Skim milk (0.1 wt% fat)	• Mini Traction Machine (MTM, PCS instruments, UK) • Ball on disk	• Load: 2 N • Contact pressure: ~100 kPa • Speed: 1,000–1 mm s^−1^ sliding-to-rolling ratio of 50%	• Upper: PDMS ball (19 mm diameter) • Lower: PDMS flat plate	Yes	• Static light scattering (Particle size) • Sensory analysis (appearance, mouthfeel, after-feel and taste) • Rheological (Viscoelasticity and dynamic oscillation)	(10)
Yogurt	• Full-fat yogurt (4.2 wt% fat) • Fat-free yogurt (0 wt% fat)						
Cream cheese	• Full fat (21.5 wt% fat) • Low fat (2.5 wt% fat)						
Milk	Milk (0.08, 2, and 5% fat)	• Discovery Hybrid Rheometer-3 (TA Instrument, Newcastle, USA) • Double ball on plate	• Load: 1 N • Temperature: 25°C • Sample: 1 ml • Speed: 0.15–750 mm s^−1^	• Upper: 2-polypropylene balls (15.88 mm diameter) • Lower: PDMS disk (40 mm diameter)	No	• Rheological (Viscosity) • Confocal laser Scanning microscopy • Fat globule size • Protein separation (SDS-PAGE) • Proximate analysis (Milk solids, fat, and protein contents) • Sensory analysis (astringency)	(11)
Milk	Pasteurized milk (0.1, 1.3, 2, 3.8, 4.9% Fat) (3.9, 3.5, 4.1, 3.6, 3.6 % protein)	Discovery hybrid rheometer, using ring on plate tribo-rheometry (TA Instrument, USA)	• Load: 1 and 2 N • Temperature: 35°C • Speed: from 0.01 to 100 s^−1^ with 20 points per decade	• Upper: Stainless steel ring • Lower: 3M™ transpore surgical tape	No	• Rheological (Viscosity) • Laser scattering (Particle Size).	(12)
Cream cheese	(0.5, 5.5, 11.6% Fat) (13.9, 11.5, 9.4% Protein)						
Pot-set yogurt	• Commercial yogurt (0.1, 1.3, and 3.8% wt fat) • Yogurt (0.1% fat) with: • Gelatin (0.5–1.5%) • Xanthan gum (0.005–0.015%) • Carrageenan (0.01–0.08%) • Modified starch (0.5–1.5%)	Discovery hybrid rheometer with ring-on-plate tribo-rheometry (TA Instrument, USA)	• Load: 2 N • Temperature: 35°C • Sample: 2 g • Speed: from 0.01 to 100 s^−1^ with 20 points per decade	• Upper: Stainless steel ring • Lower: 3M™ transpore surgical tape	No	• Texture analysis (firmness/hardness) • Syneresis analysis • Rheological (Viscosity and dynamic oscillation) • Microscopy (Distribution of fat and protein) • Sensory analysis (Thickness, Smoothness, Creaminess, Powderiness, Stickiness, Lumpiness, Oily coating, Residual coating)>	(13)
Butter	• Dairy cream (38% fat wt/wt) • Emulsifiers • Sodium Caseinate (0.5, 1.5 % wt/wt) • Tween 80 (0.5 % wt/wt)	Discovery Hybrid Rheometer and 3- ball on plate tribo-rheometry (TA Instrument)	• Load: 2 N • Temperature: 5–35 °C • Speed: 15,000 mm s^−1^	• Upper: Not specified • Lower: 3M™ transpore surgical tape	No	• Static light scattering (Particle size) • Confocal laser Scanning microscopy • Differential scanning calorimeter (Solid Fat content) • Cryo-SEM imaging • Proximate analysis (Fat, protein and moisture content) • Texture (Hardness) • Rheological (Viscosity) • Color	(15)
Stirred yogurt	• Fat content (0.1, 6, and 12/100 g) • Protein content (3.5, 4.5, and 6/100 g) • Casein to whey protein ratio • (80:20, 60:40, and 40:60)	• Rheometer (Physica MCR 301) with a tribology accessory attached • Ball on pyramid	• Load: 3 N • Temperature: 10°C • Sample: 1.5 g • Speed: 0.001–100 min^−1^	• Upper: Stainless steel ball • Lower: elastic pad made of styrene-butadiene rubber	No	• Laser diffraction spectroscopy (Particle size) • Rheological (Viscosity and dynamic oscillation) • Sensory analysis • Appearance: Grainy and viscous • Texture: Grainy, viscous, fatty mouthfeel, slimy, creamy.	(16)
Pot-set yogurt	Fat Content (0, 4.2, 9.5% wt fat)	HFRR high-frequency reciprocating rig (PCS Instrument, UK)	• Load: 2 N (Pmax = 0.25 MPa) • Temperature: 23–25°C • Time: 60 s • Reciprocating sliding: 1 mm stroke 10 Hz • Sliding Speed: Mid stroke 20 mm/s	• Upper: PDMS ball (19.8 mm diameter) • Lower: Glass microscope slide	No	None	(17)
Pot-set yogurt	Fat content (0.1, 2.0, and 4.7%)	• Mini Traction Machine (MTM2, PCS Instruments Ltd., UK) • Ball on disk	• Load: 1 N • Temperature: 35°C • Sliding and rotational speed: 1–1,000 mm s^−1^ sliding-to-rolling ratio of 50%	• Upper: PDMS ball • Lower: PDMS disk	Yes	Rheological (Viscosity, viscoelasticity, and dynamic oscillation)	(20)
Custard	Fat content (0.9, 2.7, and 6.4%)						
Thickened cream	Fat content (13, 21 and 35%)						
B-LG solutions	• Protein content (0.5, 1, 2, 4, 7, and 10% wt) • pH (3.5 and 7.0)	• Mini Traction Machine (MTM, PCS Instruments Ltd., UK) • Ball on disk	• Load: 1 N • Temperature: 25 ± 2°C • Speed: 5 mm s^−1^	• Upper: PDMS ball (18.6 mm diameter) • Lower: PDMS disk (22.5 mm diameter and 4 mm thickness)	Yes	Sensory analysis (Astringency)	(32)
Stirred yogurt	• Yogurt with added: • Extra skimmed milk powder (100 g of skimmed milk powder/500 mL) • Whey protein concentrate (50 g of skimmed milk powder + 21.38 g of whey protein/500 mL) • Extra skimmed milk powder + 2% starch • Whey protein concentrate + 2% starch	• Texture analyzer equipped with Exponent software version 3.2 (both from Stable Micro Systems, Godalming, UK) • Ball on disk	• Load: 0.27 N • Temperature: 25°C • Sliding speed: 0.1–10 mm s^−1^	• Upper: Three stainless steel balls • Lower: 1 mm thick silicone elastomer	Yes	Sensory analysis (Free-choice term)	(33)
Fat-free yogurts	• Constant protein (5%) • Lactose (6%) • Casein to whey protein ratios (80:20, 70:30, 60:40, and 50:50)	• Rheometer MCR 301 Anton Paar Physica • with a tribology accessory (T-PTD200, BC12.7, Anton Paar Physica) • Ball on plate	• Load: 3 N • Temperature: 10°C • Speed: 0.001–1,000 min^−1^ • Sample: 1.5 g	• Upper: Stainless steel ball • Lower: Rubber pads	No	• Dynamic light scattering (Particle size) • Rheology (Viscosity) • Microscopy (Microstructure) • Sensory analysis (19 textural attributes)	(34)
Milk	Skim milk to full-fat milk (0.06, 0.15, 0.3, 0.5, 0.7, 1, 2, 3, 4, 6.5, 8.68% wt fat) (3.3% wt protein)	• Mini Traction Machine (MTM; PCS Instrument Ltd., London, UK) • Ball on disk	• Load: 5 N • Temperature: 20°C • Speed: from 500 to 5 mm s^−1^	• Upper: Stain ball • Lower: 3 mm thick disk made of silicone, neoprene or Teflon	No	• Rheological (Viscosity) • Microscopy (coalescence of the fat on the surface) • Sensory analysis (categories smell/taste, mouth-feel, mouth/after-feel and after-taste/feel sensation)	(35)
Milk	• Skim milk (50% carbohydrates, 35% protein, 1.5% fat, and 4% moisture) (10% wt/wt) • Microparticulated whey protein (0.5, 3, 6, 9, 12, 15, 18, 20% wt/wt) • Commercial homogenized cream (0.5, 3, 6, 9, 12, 15, 18, 20% wt/wt) • Hydrocolloids (0.5% wt/wt)	• Rheometer (MCR 302, Anton Paar) with a tribology accessory attached • Ball- on-three-plates	• Load: 1 N • Temperature: • 25 and 37°C • Speed: 0.0447–940 mm s^−1^	• Upper: PDMS ball (radius 6.35mm) • Lower: PDMS plates (size 3 ×6 ×16 mm) • Both treated with a high-frequency generator	No	• Microscopy (Morphological characterization) • Rheological (Viscosity)	(36)
Milk	• Skim milk (0.1 g/100 ml milk fat) + Phytosterols ester (0.8, 1.2, 1.6, and 2.0/100 g) • Commercial milk (0.1, 1.3, 2 g/100 ml fat) • Commercial milk (1 g/100 ml fat) + Phytosterols ester (0.32 g/100 ml)	Discovery Hybrid Rheometer, using ring on plate tribo-rheometry (TA Instrument, USA)	• Load: 2 N • Temperature: 35°C • Speed: from 0.1 to 100 s^−1^ with 20 points per decade	• Upper: Stainless steel ring • Lower: 3M™ transpore surgical tape	No	• Color • Rheological (Viscosity) • Static light scattering (Particle size) • SPME-GC (Volatile compounds)	(37)
						• Sensory analysis (Creaminess, Graininess, Thickness/viscosity, Oily mouth coating, and Off-flavor)	
Cream cheese	• Cream cheese • 5.5% milk fat • 3.2% (wt/wt) phytosterols (emulsion or esters) • 3% (wt/wt) β-glucan • Commercial cream cheese (13.7, 24.2% fat)	Discovery Hybrid Rheometer with ring-on-plate tribo-rheometry (TA Instrument, USA)	• Load: 2 N • Temperature: 35°C • Sample: 2 g • Speed: 30–0.01 rad s^−1^ with 20 points per decade	• Upper: Stainless steel ring • Lower: 3M™ transpore surgical tape	No	• Moisture analysis • Static light scattering (Particle size) • Microscopy (Distribution of fat and protein) • Texture analysis (firmness, spreadability, adhesiveness) • Rheological (Viscosity)	(38)
Yogurt	• 4.5% (w/v) protein • Complex solutions • Fish Gelatin–Arabic gum/Xanthan gum/ κ- carrageenan • Ratios 10:0, 9:1, 7:3, 5:5, 3:7, 1:9, 0:10 (wt/ wt)	Discovery Hybrid Rheometer, using ring on plate tribo-rheometry (TA Instrument, USA)	• Load: 1 and 2 N • Temperature: 35°C • Speed: from 0.01 to 273 s^−1^ with 10 points per decade	• Upper: Stainless steel ring • Lower: 3M™ transpore surgical tape	No	• Zeta potential • Static light scattering (Particle size) • Rheological (Viscosity) • Texture analysis (Firmness and adhesiveness) • Water holding capacity	(39)
Stirred yogurt	• Skim yogurt (0.1% fat) • Full fat yogurt (3.8% fat) • Inulin (7, 8 9%) • Pectin (0.2, 0.25, 0.3%) • GOS (9.1, 11.3, 13.6%) • β-glucan (0.1, 0.2,0.3%).	Discovery Hybrid Rheometer, using ring on plate tribo-rheometry (TA Instrument, USA)	• Load: 2 N • Temperature: 35°C • Speed: from 0.01 to 100 s^−1^ with 20 points per decade	• Upper: Stainless steel ring • Lower: 3M™ transpore surgical tape	No	• Color analysis • Syneresis analysis • Texture analysis (Firmness and stickiness) • Static light scattering (Particle size) • Microscopy (Microstructure) • Rheological (Viscosity) • Sensory analysis (creaminess, astringency, thickness, smoothness, lumpiness, chalkiness, fatty feel, stickiness, oily coating, residual coating)	(40)

*B-LG, Beta-lactoglobulin; GOS, Galactooligosaccharides; PDMS, Polydimethylsiloxane; SPME-GC: Solid-Phase Microextraction followed by Gas Chromatography*.

### Stribeck Curve in Dairy Products

As mentioned before, the traditional Stribeck Curve is divided into three regimes. However, for dairy products a particular situation occurs, since more zones could be observed, and this is due to the complexity of the matrix involving different hydrocolloids. Nguyen et al. (13) described 4 zones: Zone 1: this zone is characterized by having a very narrow space between the two surfaces. So the CoF in this zone is governed by the soluble substances and small particles dispersed in the liquid whey, such as whey protein and free fat globules, which migrated from the gel matrix. And when there are a lot of small particles, the friction is gradually reduced from dry contact to a minimum value that will be the guideline for zone 2. Zone 2: the rest of the fluid (in the form of a gel) begins to enter the contact zone, gradually increasing friction. A thin film of lubrication is created between the contact surfaces that increases friction until it reaches its maximum value (this will be a second transition point, which will mark the end of zone 2 and the beginning of zone 3). Unlike the traditional Stribeck curve, here the friction is no longer a constant value but increases linearly with speed. Zone 3: the friction begins to decrease due to the fact that the lubrication film grows even more and the thickness increases. In this area, viscosity plays an important role. Zone 3 can be compared with the mixed regime in the conventional Stribeck curve. Once again the CoF reaches a minimum point and marks the start of zone 4. Zone 4: from the minimum point of friction generated in zone 3, the friction curve changes slope. The friction can reach another minimum point at the end of the mixed regime (point T3) if the fluid retains its structure, and increases again with increasing speed. Now the surfaces are completely separated because the hydrodynamic film is fully developed, this zone is the same as the hydrodynamic regime in the traditional Stribeck curve. The CoF is somewhat determined by the internal friction (or fluid viscosity) and increases linearly with speed.

On the other case, if the speed is so high that it breaks down the fluid structure, the friction can be further reduced with speed. So not only the viscosity plays an important role in this zone, but also the gel strength of the sample.

**Figure 4:**
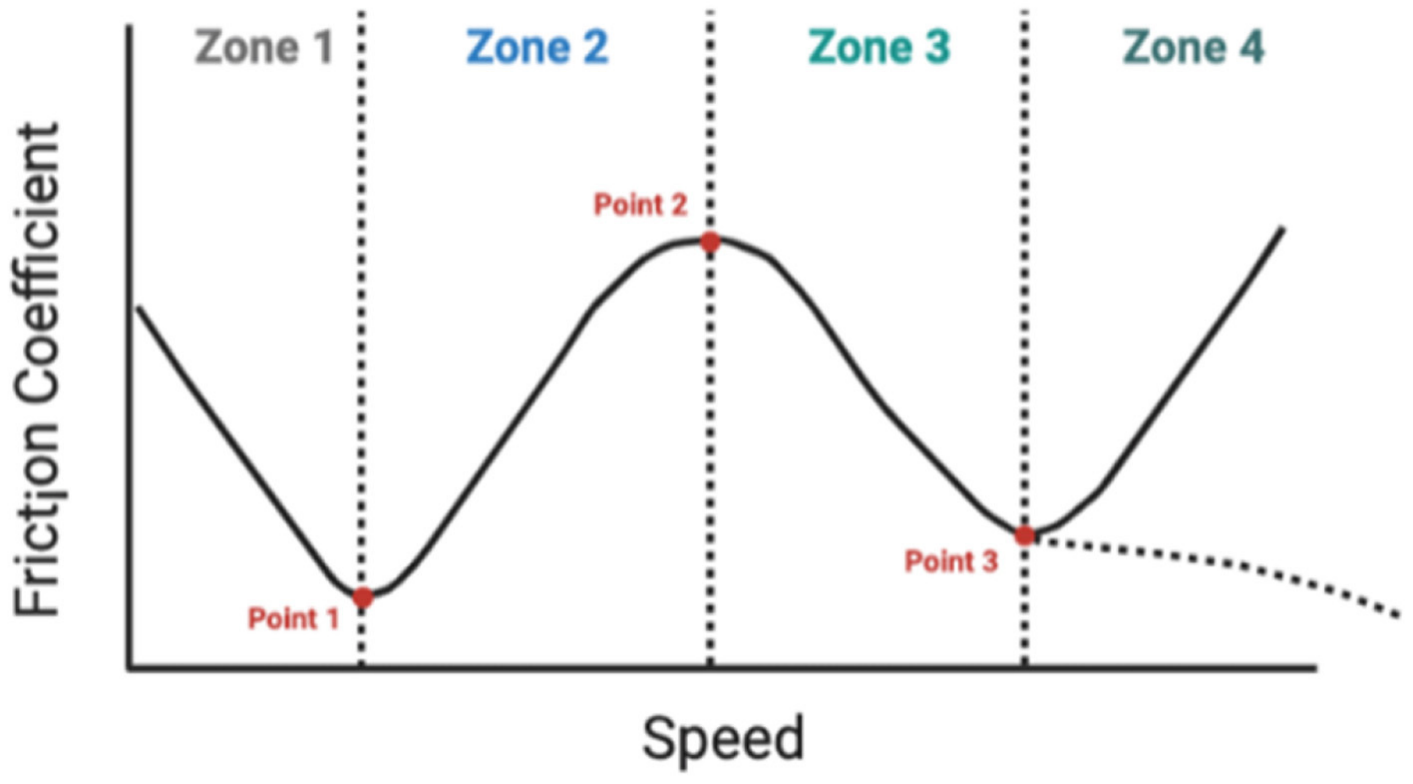
Graph of the coefficient of friction divided into 4 zones possibly observed in dairy products. Figure edited from Nguyen et al. (13).

What was described by Nguyen et al. (13) is similar to a cosine wave as can be seen in Figure 4. However, (30) in their work shows that there may be more forms of the curve in dairy products. They reported, in addition to the traditional Stribeck curve, other shapes such as: “S,” “C,” “U,” and “W.” These were obtained depending on the starter culture, the composition of the milk base (stabilizers such as starch and gellan gum) and the production process. They also mention that the physical and chemical properties such as adhesion, wettability, viscoelasticity, plasticity, hardness, and roughness of the compound played a key role in each zone/regime.

### Sensory Perception

Taste and texture are critical factors in designing and developing food to reach the final consumer's desired characteristics. The consumer evaluates these descriptions during the oral processing of different foods (7, 41). Table 2 shows the definition of the essential dairy product attributes associated with their textural description.

**Table 2:** Definitions of sensory attributes in dairy products.

**Attributes**	**Definition**	**References**
Thickness	• The degree to which the sample looks and feels thick • Mechanical property perceived when compressing the product between the tongue and the palate • Resistance to flow in the mouth before saliva modifies the sample	(13, 32, 42)
Smoothness	• The degree to which the sample feels smooth in the mouth, with an absence of lumps or granules. Assessed by swirling the sample in the mouth • Perceived smoothness of the sample squeezed between palate and tongue	(13, 32)
Creaminess	• The perceived silky smoothness of the sample. Assessed by moving the tongue parallel to the palate in circles • Silky smooth sensation in the mouth • Combined perception of fat, smoothness, and viscosity	(13, 32, 42)
Cohesiveness/Stickiness/Adhesiveness	• Degree to which the sample sticks around the mouth and coats the mouth surfaces (roof, cheeks, teeth). Assessed by pressing the sample between palate and tongue • Mechanical attribute relating the degree to which a substance can be deformed before it breaks • Degree to which the sample sticks to the teeth and palate	(13, 42)
Lumpiness/Grainy	• The degree to which large, soft lumps are perceived in the mouth. Assessed by swirling the sample in the mouth • Amount of soft lumps or graininess present in the sample	(13, 42)
Mouth coating/Residual coating	• The mouthfeel of the product once swallowed, the perception of a thin layer covering the palate • Intensity of residues left in the mouth after swallowing	(13, 32, 42)
Astringency	• Drying-out and puckering sensation that follows the consumption of particular food or drinks	(6)

One of the most important components of sensory perception is the amount and type of fat present in dairy products. Fat can be retained as globules during oral processing, released as free fat, or mixed with other food ingredients depending on the type of food. This is a crucial factor in the attributes of smoothness, creaminess and astringency, among others (43).

Nevertheless, non-fat ingredients have recently become a valuable alternative for enhancing food texture by replacing fats. For instance, ice cream can use carbohydrate-based molecules to decrease ice crystal formation, improve texture and mouthfeel, and reduce fat content (44). In the same way, proteins are fundamental to dairy products texture, hardness, and organoleptic properties. In addition, recent studies have shown insights about controlled protein aggregation, which could relate to a similar texture, creamy appearance, and consistency of full-fat products, such as whey proteins that provide fat-like functions in terms of flavor, mouthfeel, and texture in light and low-fat formulations (45, 46).

### Astringency

Astringency in dairy products is associated with fat content and the percentage and type of protein (11, 32, 43). Examples of astringent compounds are tannins, polyphenols, positively charged proteins, and polysaccharides (5, 6). The astringency attribute is believed to occur when there is an aggregation and precipitation of salivary proteins, causing saliva to lose its lubricating capacity (11). Therefore, tribology appears as a valuable tool to link this molecular phenomenon with sensory perception because astringency is described as reducing the lubrication capacity provided by saliva in the oral cavity (6).

Vardhanabhuti et al. (32) were the first to study the effect of β-Lactoglobulin (B-LG) found in whey on saliva and its relationship with astringency. In their work, they evaluated this protein at different pHs (3.5 and 7.0), depicting an increase in the CoF almost instantly when B-LG at pH 3.5 was added. The authors established that this increase could be due to a depletion of the lubricating salivary protein film and/or the complexes formed between salivary proteins and B-LG. However, more research is required to elucidate the precise mechanism. On the other hand, B-LG's CoF at pH 7 did not have relevant saliva changes, suggesting that it would have low astringency. Finally, in the same work, they compared the B-LG concentrations at pH 3.5 from 0.5 to 10.0% w/w. The results showed inconsistencies between the concentrations and CoF. The coefficient of the highest protein concentration was below the values observed for the lowest protein concentrations. It was concluded that there was no simple relationship between sensory results and tribology, suggesting that the complex and dynamic system in the oral environment plays a key role in the perception of astringency. An important aspect is that (32) did not consider the particle sizes of the sample, which is not surprising since they carried out the first tribological studies in dairy.

Additionally, (33) found a relationship between the type of protein and astringency. This study tested yogurt samples with two protein types: whey protein concentrates and skimmed milk powder. The results showed less acceptability by the consumer panel with descriptions such as graininess or astringency and a significant reduction in the CoF for samples containing whey protein. Since the authors suggest that this coefficient's reduction is due to particle size and hypothesized that this reduction could be due to the inability of the whey particle (due to its large size) to fit between the asperities of the sliding surfaces when producing a lubrication effect. These observations complement the (32) report, where B-LG in whey protein also reduced the CoF (Table 1).

Regarding milkfat, this component reduces the sensation of astringency. Li et al. (11) compared the astringent effect of ultra-pasteurization (UP) and high-temperature short-time pasteurization (HTST) in milk with <0.2, 2.0, and 5.0% fat. According to the sensory analysis with a trained panel of consumers, it was shown that UP increased the astringency of the milk compared to HTST pasteurization. However, the tribological results were inconsistent, so the relationship between friction and the astringent sensation was not fully determined. Nevertheless, the authors reported lower CoFs values associated with the high-fat samples (Table 1).

Furthermore, (47) evaluated the sensory perception in cottage cheese with 0, 1, 3, 8, and 11% fat content, reporting that astringency and bitterness perceived by an experienced panel decreased as the fat increased. In this study, tribological analyses were not performed. However, (14) performed a tribological analysis in cream cheese with different fat concentrations (0.5, 5.5, 11.6% w/w) (Table 1), determining an inversely proportional relationship between fat percentage and CoF. The authors stated that low-fat cream cheeses were associated with higher CoF, firmer texture and reduced spreadability. They attributed the higher CoF values to a thin layer of fat between the surfaces, discussed later. There are still many analyses for other astringent substances to describe the relationship between the precipitation of proteins from saliva and the astringency of those substances (11). It cannot be assured that the CoF will increase for all of them because other aspects are involved, such as the particle size as previously commented and layers formed on the surfaces, as will be discussed in Section Dairy Products, Sensory Perception, and Tribology.

### Smoothness and Creaminess

Smoothness is associated with a movement in both surfaces (upper and lower) inside the mouth (48), whereas creaminess focuses on the movement of the tongue parallel to the palate in circles (34). In dairy products, fat is the most crucial factor related to these attributes, being then important to understand its relationship with CoF. One of the first works to associate CoF with fat and the sensory attributes of creaminess was done by Chojnicka-Paszun et al. (35), who evaluated the sensory perception of homogenized and pasteurized milk with fat content between 0.06 and 8.7%, correlating CoF with viscosity values. An MTM tribometer with a ball was used for the disk test, in which three types of 3 mm thick disks surfaces were compared: silicone, neoprene, or Teflon (Table 1). The authors reported correlation coefficients between creaminess and CoF of −0.92 and −0.94 for neoprene and silicone, respectively, when the milk's fat content was between 0.06 and 4.0%. This effect was attributed to forming a thin layer because of the coalescence of fat globules on the surfaces. This layer could be observed at low speed, above the fat content of 1 and 2% in silicone and neoprene, respectively. The high correlation coefficient for silicone rubber may indicate that it is the best material to represent the oral environment for homogenized and pasteurized milk tested under the described conditions. Fused fat droplets were separated at high speeds, causing an increase in CoF (35).

Selway and Stokes (20) studied the potential of soft tribology to differentiate commercial dairy products with similar rheological properties using an MTM with PDMS. They worked with a different fat percentage in custard (0.9, 2.7 and 6.4%), pot-set yogurt (0.1, 2.0 and 4.7%), and thickened cream (13, 21 and 35%). In their analysis, the function of pre-absorbed saliva was contemplated (Table 1). Results indicated that there were no significant differences in the bulk rheological profile in almost all the products, except high-fat custards. Due to their weak gel structure, the products exhibited a non-Newtonian flow behavior and some solid-like properties. However, the differences found in the CoF were interesting. In general, all low-fat products exhibited higher CoF than medium and high-fat counterparts. In yogurt, as in thickened cream, the medium and high-fat content samples had very similar CoFs. This could be attributed to fat being an excellent lubricant and reduced adhesion. This work by Selway and Stokes (20) demonstrated that tribology is a valuable tool for dairy characterization and food differentiation because, despite the similar bulk rheological profiles of the products, they exhibited unique tribological properties. Laguna et al. (10) evaluated the relationship between the rheological and tribological properties with the sensorial characteristics of commercial milk, yogurt and cream cheese (full fat and free fat versions) using an MTM tribometer with a PDMS flat plate and a 19 mm diameter ball. Two fat concentrations for each product were used: milk (3.6 and 0.1%), yogurt (4.2 and 0 %), and cream cheese (21.5 and 2.5%) (Table 1). In the sensorial results, untrained panelists were able to distinguish between the full fat and fat-free/low samples of the three dairy products, whereas the product's flow behavior (in the presence and absence of saliva) was similar, only with cream cheese depicting a moderately higher G' in full-fat samples. In the case of the tribological results, at low entrainment velocities 1–100 mm s^−1^, CoFs, as in sensorial results, depicted differences in full-fat yogurt and cream cheese compared to fat-free/low-fat alternatives. The only exception was the traction coefficients for milk.

Sonne et al. (16), besides analyzing different fat contents (0.1, 6, 12/100 g) in stirred yogurt, also evaluated the effect of protein content (3.5, 4.5, 6.0/100 g) with different ratios of casein/whey proteins (80:20, 60:40, 40:60) on lubricating properties and correlated the results to properties as creaminess. The authors used a stainless-steel ball as a palate and a styrene-butadiene rubber elastic pad simulating the human tongue. Although different tribological methods and conditions were used, their conclusion supports (35) idea regarding fat content and coalescence in which increasing fat was related to a decrease in CoF and the formation of an interfacial film between surfaces. Higher friction discrimination was observed at speeds below 10 mm s^−1^. Moreover, CoF decreased with increasing protein levels and when reducing whey proportion, suggesting that the content and type of protein also affected important attributes such as creaminess and smoothness. The authors concluded that the in-mouth creaminess of yogurt is a multi-sensory experience and related to the combination of rheological, tribological and particle size characteristics. They were able to deduce three important features to produce a fat-reduced yogurt with comparable in-mouth creaminess to a full-fat yogurt: (1) small particle size (d_90, 3_ ≤50 μm); (2) high viscosity (shear stress ≥80s^−1^, at 100 s^−1^) and (3) low friction (friction coefficient ≤ 0.3, at 1 mm s^−1^).

Adding alternative structural compounds such as hydrocolloids (13), polymeric thickening agents (49), microparticulate whey protein (36), phytosterols (37, 38), and microbubbles (50) could improve the creaminess and smoothness without using extra fat, achieving healthier product options. Recently, tribological research has focused on the effects of some hydrocolloids, such as xanthan gum, gelatin (13), fish gelatin (39), carrageenan (9, 13), and starch (9, 13, 33) (Table 1). Nguyen et al. (13) researched and compared yogurts with three ratios of fat (0.1, 1.3, and 3.8%) with skim yogurt (0.1% fat) added with gelatin (0.5, 1.0, 1.5%), modified starch (0.5, 1.0, 1.5%), xanthan gum (0.005, 0.010, 0.015%), and carrageenan (0.01, 0.04, 0.08 %). This study established one methodology for tribological studies in dairy products, using a Discovery Hybrid Rheometer, with a ring-on-plate on a rough plastic surface of 3M Transpore Surgical Tape as the lower surface. Samples with xanthan gum, starch, and carrageenan exhibited a higher CoF than the control (skim yogurt), so they were less creamy. These samples were described by consumers as lumpy and chalky, while counterparts produced with gelatin gave the greatest resemblance to a full-fat yogurt. The sample containing the hydrocolloid showed similar thickness, smoothness, and creaminess and had a comparatively lower CoF than full-fat yogurt.

In addition to the hydrocolloids mentioned above, (40) also investigated the effects on the CoF and sensory perception when adding functional ingredients such as inulin (7, 8, and 9%), pectin (0.2, 0.25, and 0.3%), galactooligosaccharides (GOS, 9.1, 11.3, and 13.6%), and β-glucan (BG, 0.1, 0.2, and 0.3%) as a replacement for fat in skim stirred yogurt (0.1% fat, Table 1). The results were compared with full-fat stirred yogurt without any addition of ingredients. The overall conclusions indicated that none of the ingredients significantly improved the sensory attributes of skim yogurt, which makes sense since the CoFs did not have significant changes compared to the CoF of the full-fat yogurt. In fact, in samples supplemented with pectin and BG, the smoothness and creaminess were reduced. They caused an undesirable mouthfeel of lumps and coatings due to the presence of large gel particles, probably due to the poor solubilization or inadequate incorporation of the ingredients during yogurt manufacturing. However, the authors did not discuss this possibility, and they determined that GOS and inulin were the best ingredients in this group, as they showed a lower CoF and did not affect the texture, gel particle size, rheological and sensory characteristics compared to pectin and BG. Apparently, single fat replacers cannot completely substitute or fulfill all the fat functionality. Therefore, the combination of two or more fat substitutes was the subject of an investigation by Ningtyas et al. (38) since it represents a promising option to compensate for the lack of texture and sensory attributes. They analyzed the effect on the textural, microstructural, and lubrication properties of five combinations of phytosterols (PS, emulsified, and esterified form) and β-glucan (BG) reduced-fat cream cheese. The final composition of the samples was 5.5% milk fat, 3.2% (w/w) PS, and 3% (w/w) BG (Table 1). The results were compared with low-fat cream cheese (LFCC) with any fat replacer and with commercial cream cheeses: high-fat (24.2% fat, P1) and reduced-fat (13.7% fat, P2).

General conclusions showed that BG combined or alone increased the viscosity similarly to a high-fat cheese. Moreover, it showed relatively high moisture content, firmness, and adhesiveness due to its ability to bind water. The addition of PS improved the spreadability of cream cheeses, improved lubrication, and reduced CoF values. However, it did not provide any significant effect on viscosity compared to P2 and LFCC. Recently, a similar study was carried out on milk by Goh et al. (37) (Table 1). In this work, the addition of PS ester (0.8, 1.2, 1.6, and 2.0 /100 g) into skim milk (0.1 g/100 mL milk fat) was evaluated and compared to commercial milk (0.1, 1.3, 2 g fat/100 mL, and 1.3 g fat/100 mL with 0.3 g PS/100 mL). This study concluded that the viscosity and lubrication resulting from milk enriched with PS are comparable with commercial products at the same total fat content. As the PS ester concentration increased, CoF decreased, while the size of the fat globules and the viscosity increased.

## Relationship of Adsorption Studies With Tribology and Dairy Products

Tribology has certainly attracted the attention of food research. Although many works related to the sensory aspects of foods have already been reported, there is still much to investigate in other areas to guide the decision-making process when foods are being formulated or reformulated. As described earlier, fat substitution aimed to maintain the functionality and sensory properties in dairy products is among the main interests worldwide. Kew et al. (51) discussed the most viable options to replace fat in food products, including protein concentrates from animal and plant and microparticulated forms of proteins, concluding that whey protein is the best and most commonly used for substitution. Following this statement, researchers are currently investigating replacing whey protein in dairy products with vegetable protein since it could have great potential in the industry, but its understanding, application and characterization are still limited. Omrani Khiabanian et al. (52) studied the replacement of whey protein with pea protein in feta cheese to understand the effect on the chemistry, rheology, texture, and microstructure. They varied the proportions of both proteins to reach 12% of the total composition and concluded that by increasing pea protein above 3%, feta cheese began to have undesirable sensory properties, especially flavor, and texture.

Additionally, by gradually increasing the vegetable protein concentration, the hardness, cohesiveness, springiness, gumminess, and chewiness of the cheese were reduced. Although this research did not include tribological analyses, it was the beginning to understand the effect of this vegetable protein on dairy. Recently, (53) hypothesized that rheology, lubrication, and adsorptive properties of pea protein could cause these unpleasant effects on food texture. Adsorption is described as an augmentation in the concentration of a substance, known as adsorbate, at the interface of a liquid or gas layer, because of the operation of surface forces (54). Once adsorbed, the adsorbate resides in a surface known as adsorbent, being adsorption classified according to two main mechanisms: physisorption and chemisorption (42). The first one occurs when binding interactions are <40 kJ/mol, and adsorbate-adsorbent interactions mainly are associated with van der Waals forces, whereas chemisorption is the adsorption mechanism with binding interactions higher than 40 kJ/mol and intramolecular association governed by covalent binding (42, 54). The adsorption studies in soft tribology allow a better understanding of the boundary lubrication regime, where CoF is dependent on non-hydrodynamic characteristics.

There are still few studies of friction in dairy foods; nevertheless, the techniques for investigating adsorption have attracted attention as a complementary tool for tribological analysis. Among the experimental techniques reported to study the boundary regime are those focused on solid surfaces in contact with the lubricant or food, used to study the structural, physical and chemical aspects from macroscopic to atomic-scale (54). The instruments used are optical, scanning tunneling, atomic force microscopes, and stylus profilometer. To measure the amount of mass and thickness of adsorbate are Quartz Crystal Microbalance (QCM) and the ellipsometer (54). The most widely reported study on the adsorption phenomena in food is the QCM with Dissipation Monitoring (QCM-D), which measures adsorption in real-time.

Moreover, QCM-D can record the frequency and energy dissipation changes as a function of time to monitor adsorption (53, 55, 56), providing information about the adsorption kinetics, mass and viscoelasticity of the adsorbate. Zembyla et al. (53) used PDMS-coated sensors on this equipment to mimic the human oral surfaces besides the gold-coated alternatives. Zembyla et al. (53) compared the rheological, lubrication, and adsorbent properties of whey and pea protein dilutions with and without heat treatment in the presence or absence of salivary mucin on the surface of the PDMS sensors. The QCM-D results showed that pea protein adsorbed twice as much on PDMS surfaces and forms a slightly more viscous film than whey protein. Pea protein can be better adsorbed in salivary mucins than whey protein, forming films with similar viscoelastic properties. The high adsorption capacity of pea protein resulted in better lubrication for concentrations lower than 10 mg/ml, which was not observed for whey protein. However, when increasing the concentration to 100 mg/mL, the pea protein tended to form aggregates which negatively affected the lubrication and increased CoF, while whey protein improved lubrication. This demonstrates that whey protein needs higher concentrations to saturate the contact surfaces than pea protein. In conclusion, replacing whey protein with high pea protein concentrations could negatively affect the sensorial characteristics of foods developed with this ingredient.

Omrani Khiabanian et al. (52) reported that feta cheeses with low concentrations of pea protein were texturally more compact and hard, which could mean an increase in the CoF. The most recent study on the lubrication and adsorption of alternative proteins for fat substitutes was carried out by Kew et al. (57), evaluating dilutions of five types of proteins (1) Whey protein isolate (WPI), (2) Pea protein concentrate (PPC), (3) Potato protein isolate (PoPI), (4) Insect protein concentrate (IPC, *Alphitobius diaperinus*) and (5) Lupine protein isolate (LPI). The QCM-D results demonstrated that the final film viscoelasticity was higher for IPC followed by PPC, LPI, WPI, and PoPI, respectively, revealing that PoPI and WPI formed the most rigid layers, with low CoF, whereas the most viscous films as PPC led to high friction coefficient. At concentrations of 5%, all proteins showed effective lubrication, but when increasing the concentration to 10%, CoF of LPI, PoPI, and IPC increased, while WPI showed the lowest boundary friction coefficient and the best lubrication performance. Perhaps it is advisable to use alternative proteins in low concentrations for food development. However, to verify this information, it is necessary to study the direct effect of these proteins in the food system, including the adsorption studies, which can be useful better to understand lubrication and friction in the boundary regime. With the mixed regime, this section is where CoF tends to correlate with a range of sensory properties such as smoothness, slipperiness, and pastiness (57). The use of specific methodologies and instruments to study adsorption is an opportunity area not fully explored yet.

## Conclusions

In recent years, several investigations have been carried out dealing with tribology and its relationship with the sensory perception of foods, especially smoothness, creaminess, and astringency. There is a constant relationship in dairy products, where the higher the fat in the food, the greater the smoothness and creaminess, thus reducing the coefficient of friction generated during the tribological analysis and, in turn, reducing the sensation of astringency in the oral cavity. However, it has also been observed that dairy proteins can produce a sensory perception of astringency when these interact with salivary proteins because both have a different charge that can leads to an increase in friction and/or the loss of saliva lubrication if these are at low pH. It is important to mention that this document has not added other sensory attributes such as ropiness, chalkiness, powderiness and others due to the lack of research that studies them explicitly. Due to their components (fat, protein, carbohydrates, stabilizers), dairy products represent an interesting matrix for tribological studies, in which multiple factors come into play since they can cause high variability in the results. In previous review articles on lubrication and food, great emphasis was placed on determining exactly the best system to use for the type of food wanted to study. For example, the type of tribometer and surface material to obtain more reliable results and if using saliva provides more realistic scenarios. Today, we discussed adsorbent properties as the newest topic that complements tribology. So far, there are not still many studies on adsorption and tribology for dairy products. However, it is expected that just as the particle size is essential for friction analysis, so will adsorption. To this day, tribological results have offered significant advances in sensory analysis not only for dairy products but for the food industry in general.

Furthermore, it proves to be a promising predictive tool that supports and complements conventional systems (texture and rheology) in new product development. However, despite the evident advantages of tribology, its use in industrial and commercial applications has some important challenges because it cannot be applied in solid foods, and the analytical equipment is expensive. Besides, instruments, measurements, sample preparation, and personnel training are important parameters for obtaining reliable results that yield case-specific data. Therefore, the use of tribometers with convectional systems as rheometers and trained sensorial panels is needed.

Currently, few food companies have integrated tribology as one of their tools for food analysis. However, in a short time, more institutions are likely to become aware of this technique and its several applications for decision making in the development of new and healthy food products contributing to research and a better understanding of the use of soft tribology in the food industry.

## Author Contributions

BC-P collected data, drafted the manuscript, and adjusted information same that AS-R, who besides reviewed and edited the manuscript. DM and SS-S reviewed and edited the manuscript. RE-C collected data and drafted the manuscript. Finally, CC-H conceptualized the article, drafted, reviewed, and edited the manuscript. All authors contributed to the article and approved the submitted version.

## Funding

This research was supported by the Research Chair Funds from Centro de Investigación y Desarrollo de Proteínas (CIDPRO), The Institute for Obesity Research (Tecnológico de Monterrey Campus Monterrey) and Beatriz Corvera's postgraduate fellowships by Consejo Nacional de Ciencia y Tecnología (CONACyT), and Tecnológico de Monterrey.

## Conflict of Interest

The authors declare that the research was conducted in the absence of any commercial or financial relationships that could be construed as a potential conflict of interest.

## Publisher's Note

All claims expressed in this article are solely those of the authors and do not necessarily represent those of their affiliated organizations, or those of the publisher, the editors and the reviewers. Any product that may be evaluated in this article, or claim that may be made by its manufacturer, is not guaranteed or endorsed by the publisher.
